# Machine Learning in Adolescent Mental Health: Advanced Comorbidity Analysis and Text Mining Insights

**DOI:** 10.3390/healthcare13172159

**Published:** 2025-08-29

**Authors:** Dafni Patsiala, Konstantinos Bolias, Fani Passia, Georgios Feretzakis, Athanasios Anastasiou, Yiannis Koumpouros

**Affiliations:** 1 Ocelot Special Psychosocial Intervention Unit—F.C.T.E Society, 111 41 Athens, Greece; 2 School of Science and Technology, Hellenic Open University, 263 31 Patra, Greece; 3 Biomedical Engineering Laboratory, National Technical University of Athens, 157 72 Zografou, Greece; aanastasiou@biomed.ntua.gr; 4 Digital Innovation in Public Health Research Lab, University of West Attica, 115 21 Athens, Greece; ykoump@uniwa.gr

**Keywords:** adolescent mental health, machine learning, association rule mining, cluster analysis, topic modeling, ICD codes, comorbidity

## Abstract

**Background**: Justice-involved adolescents exhibit high rates of mental health disorders with complex comorbidity patterns. Understanding these patterns is crucial for developing targeted interventions in this vulnerable population. **Methods**: We applied multiple machine-learning techniques to electronic records from 124 justice-involved adolescents (11–21 years; mean = 15.7 ± 1.9). Analyses included association rule mining, K-Means clustering with t-SNE visualization, and topic modeling of clinicians’ recommendation notes. **Results**: Hyperkinetic disorders (F90.0/F90.1) and family-stress factors (Z63.5) together accounted for approximately 45% of all ICD-10 entries. A four-cluster K-Means solution built on age + F-codes alone showed weak separation (silhouette = 0.044), whereas adding Z-codes markedly improved cohesion (silhouette = 0.468) and isolated a distinct hyperkinetic–family-stress subgroup. Association-rule mining returned one robust rule, F81 → F90.0 (support = 0.048, confidence = 0.46, lift = 1.59), underscoring the frequent co-diagnosis of learning and attention-deficit disorders. Topic modeling of clinicians’ recommendation notes recovered five coherent intervention themes—vocational guidance, parent counseling, psycho-education, family psychotherapy, and psychiatric follow-up—which aligned closely with the data-driven clusters. **Conclusions**: These findings demonstrate how routine clinical data can reveal actionable comorbidity profiles and guide tailored interventions for complex adolescent mental-health presentations.

## 1. Introduction

Adolescent mental health represents a critical public health priority with far-reaching implications for individual development, family systems, and societal well-being [1]. Recent global estimates suggest that 10–20% of adolescents experience mental health conditions, with the majority remaining undiagnosed and untreated [2]. The adolescent period, characterized by significant neurobiological, psychological, and social transitions, creates a unique vulnerability to the emergence of mental health disorders [3]. Simultaneously, this developmental stage offers a crucial window for intervention, as effective treatment during adolescence can alter negative trajectories and significantly improve long-term outcomes [4,5].

Mental health disorders in adolescence rarely present as isolated conditions. Rather, they typically manifest as complex constellations of symptoms and comorbidities that span diagnostic categories, challenging traditional taxonomic approaches [6]. This complexity is further compounded by the influence of psychosocial factors, including family dynamics, educational environments, and socioeconomic circumstances, which interact bidirectionally with neurobiological vulnerabilities [7]. For instance, attention-deficit hyperactivity disorder (ADHD) frequently co-occurs with conduct disorders, learning disabilities, and anxiety disorders, creating heterogeneous clinical presentations that require nuanced assessment and individualized intervention strategies [8].

The recognition of this heterogeneity has prompted calls for a paradigm shift in psychiatric nosology and treatment planning, moving from categorical diagnoses toward dimensional, data-driven approaches that better capture the diversity of clinical presentations [9,10]. However, implementing such approaches in routine clinical practice presents substantial challenges, as clinicians must integrate and interpret vast amounts of multidimensional data to identify meaningful patterns that can inform treatment decisions [11].

Recent advances in computational methods, particularly machine learning techniques, offer promising tools for addressing these challenges [12,13]. These approaches can process complex, high-dimensional data to uncover latent patterns and relationships that may not be immediately apparent through traditional clinical assessment [14]. In the context of adolescent mental health, machine learning has been applied to identify subtypes of depression [15], predict treatment response [16], and detect early warning signs of psychosis [17], demonstrating the potential of these methods to enhance diagnostic precision and treatment personalization.

Association rule mining, a data mining technique designed to identify frequent patterns and interesting relationships between variables, has shown particular utility in discovering comorbidity patterns in psychiatric populations [18,19]. This approach can reveal frequently co-occurring conditions, potentially illuminating shared etiological pathways or risk factors that might inform integrated treatment approaches [20]. For example, Castro et al. [21] used association rule mining to identify clinically meaningful comorbidity patterns in pediatric mental health that corresponded to distinct treatment needs and outcomes.

Complementing association rule mining, clustering techniques such as K-means offer powerful methods for identifying homogeneous subgroups within heterogeneous patient populations [22]. By grouping patients based on similarity across multiple features, clustering can reveal natural subgroups that may correspond to distinct pathophysiological mechanisms or treatment response profiles [15]. Visualization methods like t-Distributed Stochastic Neighbor Embedding (t-SNE) further enhance clustering by projecting high-dimensional data into lower-dimensional spaces while preserving local relationships, making complex patterns more interpretable for clinical applications [23,24].

Alongside diagnostic data, the free-text clinical notes generated during routine care contain rich, nuanced information about patient presentations and treatment recommendations [25]. Text mining and natural language processing techniques can extract valuable insights from these unstructured data sources, identifying patterns in clinical reasoning and treatment planning that may not be captured in structured diagnostic codes [26,27]. Topic modeling approaches such as Non-negative Matrix Factorization (NMF) have successfully identified meaningful themes in psychiatric clinical notes, offering insights into treatment decision-making processes [28,29].

Despite these advances, significant gaps remain in the application of machine learning to adolescent mental healthcare. Most studies have focused on adult populations or specific disorders, with less attention to the unique developmental considerations and complex comorbidity patterns characteristic of adolescent presentations [30,31]. Furthermore, few studies have integrated multiple analytic approaches to simultaneously examine diagnostic patterns, comorbidity relationships, and treatment recommendations, limiting our understanding of how these domains interact in real-world clinical contexts [32].

Recent studies have successfully applied integrated machine learning approaches to mental health data. For instance, association rule mining has been combined with clustering to identify psychiatric comorbidity patterns that predict treatment outcomes, while topic modeling of clinical notes has revealed intervention themes aligned with diagnostic clusters. The complementary nature of these methods is crucial: association rules identify frequent co-occurrences, clustering reveals natural patient groupings, and topic modeling extracts treatment patterns from unstructured text. This multi-method approach provides a more comprehensive understanding than any single technique alone, as structured diagnostic codes capture formal classifications while free-text notes contain nuanced clinical reasoning and contextual factors often missing from coded data.

Additionally, existing research has predominantly utilized data from highly controlled research settings, which may not reflect the messy reality of routine clinical practice [11]. There is a pressing need for studies that apply sophisticated analytical methods to naturalistic clinical data, demonstrating how machine learning can enhance real-world assessment and intervention planning [33]. Such approaches must balance methodological rigor with practical utility, producing insights that are both statistically sound and clinically actionable [34].

Particularly in the context of adolescent delinquency, where mental health issues frequently intersect with legal involvement, there is a critical need for improved methods of identifying high-risk individuals and tailoring appropriate interventions [35,36]. Justice-involved youth exhibit disproportionately high rates of mental health disorders, with complex comorbidity patterns that often include both internalizing and externalizing conditions [37,38]. Effective intervention for this population requires a nuanced understanding of how diagnostic patterns relate to psychosocial contexts and treatment needs [36], making it an ideal target for data-driven, integrative approaches.

In this study, we apply multiple machine learning techniques—association rule mining, K-means clustering, and topic modeling—to uncover deeper patterns in a dataset of 124 delinquency-referred adolescents. Our approach integrates analyses of ICD-10 diagnostic codes and clinicians’ free-text recommendations to address three key questions: (1) What are the most prevalent diagnostic patterns and comorbidities in this population? (2) Can we identify distinct, clinically meaningful subgroups based on diagnostic profiles and demographic characteristics? (3) How do treatment recommendations align with these diagnostic patterns and subgroups?

Beyond traditional epidemiological approaches, recent advances in machine learning have enhanced the detection of complex patterns across diverse domains. Transfer learning approaches, for instance, have demonstrated effectiveness in adapting machine learning models across different digital health sensing applications, enabling knowledge transfer from one sensing modality or patient population to improve performance in related clinical contexts [39]. These examples underscore how algorithms can extract subtle, context-dependent patterns from high-dimensional data. In adolescent psychiatry, similar computational approaches can reveal hidden comorbidity structures, guiding early intervention and more precise resource allocation.

By examining these questions through complementary analytical lenses, we aim to demonstrate how data-driven methods can enhance our understanding of complex adolescent presentations and inform more personalized intervention approaches. Our findings suggest that hyperkinetic disorders frequently co-occur with family stress factors, forming a distinct subtype that requires integrated treatment approaches addressing both neurobiological vulnerabilities and family dynamics. Additionally, our topic modeling reveals coherent themes in treatment recommendations that align with identified diagnostic patterns, suggesting an implicit understanding among clinicians of the relationship between comorbidity profiles and intervention needs.

These insights highlight the potential of machine learning techniques to formalize and extend clinical intuition, offering decision support tools that can help mental health professionals identify high-risk patterns and tailor interventions accordingly. By integrating structured diagnostic data with unstructured clinical notes, our approach provides a more comprehensive picture of adolescent mental health presentations than either source alone could provide, illustrating the value of mixed-method analytic strategies in clinical research.

## 2. Materials and Methods

### 2.1. Dataset Description

The final analytic sample comprised 124 justice-involved adolescents aged 11–21 years (mean = 15.7, SD = 1.9). Systematic sex/gender data were missing from many records and were therefore excluded from the modeling pipeline. Each individual carried one or more ICD-10 diagnostic codes—psychiatric (“F-” prefix) and psychosocial/contextual (“Z-” prefix). Adolescents had a mean of 1.9 codes (SD = 1.3; range = 1–7). A free-text clinician-recommendation note was available for every case and was included in the text-mining analyses.

### 2.2. Data Preprocessing

Prior to analysis, several preprocessing steps were implemented. ICD codes were extracted from the diagnostic text using regular expressions, and clinician recommendations were standardized to enable text mining. Where data contained missing values, particularly for age calculations, median imputation was performed to maintain dataset integrity.

### 2.3. Identification and Visualization of Top ICD Codes

We split each record’s ICD list, counted every code, ranked the totals, and graphed the 20 most frequent entries. The bar chart visualization enabled clinicians to instantly identify that hyperkinetic disorders (F90.0/F90.1) and family-stress Z-codes dominated, together accounting for approximately 45% of all codes. Because the underlying tally (64 unique codes) is refreshed automatically, the same routine can be rerun each quarter to track diagnostic drift. The minimum support threshold of 4% was selected to capture clinically meaningful patterns affecting at least 5 patients in our cohort, balancing statistical significance with practical relevance. The confidence threshold of 40% ensures that identified associations occur frequently enough to inform clinical decision-making. These parameters align with established psychiatric data mining studies where rare but important comorbidity patterns must be detected.

### 2.4. K-Means Clustering and t-SNE Dimensionality Reduction

Once the most prevalent ICD-10 codes were tabulated, we searched for latent patient subgroups. Each adolescent was represented by a single feature vector that contained:

1. age (z-scored);
2. one-hot indicators for every ICD-10 F-code; and
3. one-hot indicators for every ICD-10 Z-code.

The high-dimensional sparse feature space (65 dimensions with many binary indicators) poses challenges for clustering stability. To address this, we standardized continuous variables and evaluated multiple cluster validity indices beyond silhouette scores, including within-cluster sum of squares and gap statistics. The inclusion of Z-codes was critical as preliminary analyses using only F-codes yielded poor cluster separation, highlighting the importance of psychosocial context in identifying meaningful patient subgroups. (Preliminary trials that omitted the Z-codes are reported below for comparison.)

1. Model search. K-Means was selected for its speed and interpretability. We trained models for k = 2–8 with 10 random starts and 300 iterations each. Both the elbow plot of within-cluster sum-of-squares (WCSS) and the peak average silhouette width favored k = 4.
2. Impact of contextual (Z) information. Using only age + F-codes produced weak cohesion (mean silhouette = 0.044; per-point range −0.19–0.27), indicating substantial overlap between clusters. Adding Z-codes preserved cluster geometry but raised the mean silhouette to 0.468, confirming that psychosocial context is essential for separating latent subgroups.
3. Visualization. To examine cluster topology, the 65-dimensional feature space (1 age + 42 F + 22 Z variables) was projected to two dimensions with t-SNE (perplexity = 30, max_iter = 1000, random_state = 42). This stochastic neighbor-embedding preserved local neighborhoods, enabling visual inspection of subgroup boundaries.
4. Parameter choices. The chosen perplexity value of 30 lies within the recommended 5–50 range for datasets of this size; lower values fragmented obvious neighborhoods, whereas higher values over-smoothed cluster boundaries. A fixed random_state ensured reproducibility. Silhouette scores were calculated for every observation—not only the overall mean—so that low-scoring “boundary” cases could be flagged for clinical review.

All analyses were implemented in Python 3.11.9 with pandas 2.2.2 for data handling [40], scikit-learn 1.4.0 for clustering and scaling [41], and the scikit-learn t-SNE wrapper (after van der Maaten and Hinton [23]). Figures were created with Matplotlib 3.9.2.

## 3. Results

### 3.1. Demographic and Diagnostic Patterns

The analytic cohort consisted of 124 adolescents aged 11–21 years (mean = 15.7, SD = 1.9; range = 11.3–20.6). Systematic sex/gender information was unavailable in the source records and was therefore excluded from quantitative analyses.

Hyperkinetic disorders and family-related psychosocial factors again dominated the ICD-10 profile. The most frequent codes were F90.0 (hyperkinetic disorder; 35 occurrences), Z63.5 (family disruption by separation/divorce; 24), F90.1 (other hyperkinetic disorders; 21), Z62.2 (institutional upbringing issues; 14), F81 (specific developmental disorders of scholastic skills; 12), Z62 (other problems related to upbringing; 11) and F84.0 (childhood autism; 8). Collectively, these seven codes represented just over half of all diagnoses recorded in this justice-involved sample, with the hyperkinetic–family-stress triad (F90.0/F90.1 + Z63.5) alone accounting for 44.7% of the total.

**Figure 1:**
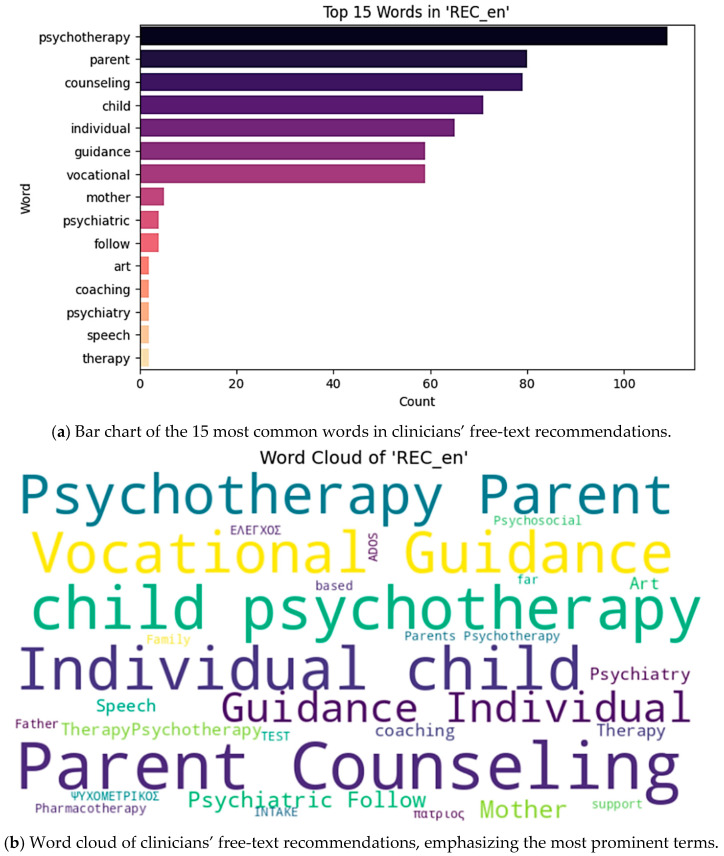
(**a**) Frequency of the 15 most common words and (**b**) word-cloud representation of clinicians ‘recommendation corpus. As a complementary view of the same corpus, we generated a word cloud to highlight relative term prominence. Larger words correspond to higher frequency in the recommendation texts.

To visualize the distribution of the most frequent terms in clinical recommendations, we present both a bar chart (Figure 1a) and a word cloud representation (Figure 1b). These figures illustrate the prominence of family-centered and vocational interventions in the clinical management of this population.

### 3.2. Comorbidity Patterns

**Figure 2:**
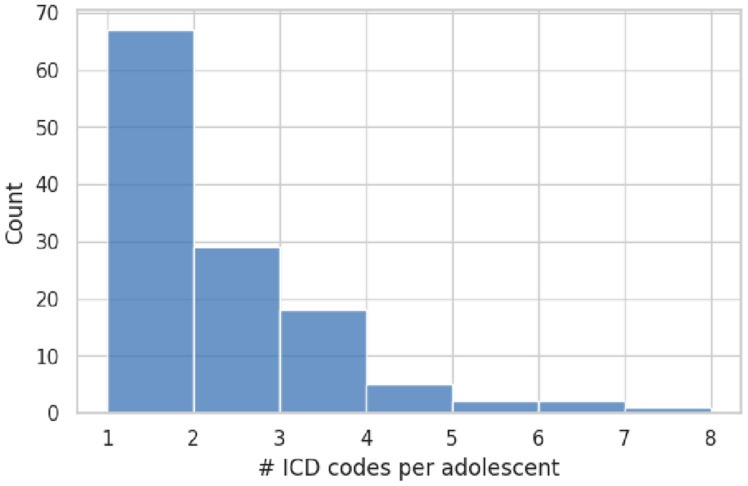
Distribution of the number of ICD-10 diagnoses per adolescent (n = 124). Most adolescents carried one or two diagnoses, with progressively fewer cases having three or more codes, and only a small tail up to seven concurrent diagnoses.

Exactly 44.4% of the sample (55/124 adolescents) carried two or more ICD-10 codes, while the remaining 69 individuals (55.6%) had a single recorded diagnosis. The distribution of diagnostic counts is shown in Figure 2. The average diagnostic load was 1.9 codes (SD = 1.3; range = 1–7). Age was not related to diagnostic complexity (Pearson r = −0.05, *p* = 0.60).

Association-rule mining. The one-hot-encoded ICD matrix was analyzed with the apriori algorithm (minimum support = 0.04, confidence = 0.40, lift > 1.0). Only one rule met these criteria:

{F81} → {F90.0} (support = 0.048, confidence = 0.46, lift = 1.59).

Thus, nearly half of all adolescents with a specific learning-disorder code (F81) were also diagnosed with hyperkinetic disorder (F90.0), underscoring the well-known co-occurrence of scholastic skill deficits and ADHD-like symptomatology in justice-involved youth.

No additional multi-item rules survived the support and confidence thresholds, indicating that other comorbidity combinations were either too infrequent or too heterogeneous to emerge as stable patterns in this dataset.

### 3.3. Cluster Analysis Results

We fitted K-Means models for k = 2–8 on the age + F-code matrix. The elbow curve and mean silhouette width both supported a four-cluster solution. Using F-codes only, the average silhouette was low (0.044; per-point range −0.19 to 0.27; median −0.04), indicating substantial overlap. Adding Z-codes retained the same geometry but raised the silhouette to 0.468, confirming that psychosocial context improves separation.

The four centroids assigned the 124 adolescents to clusters of 51 (Cluster 0, 41.1%), 48 (Cluster 1, 38.7%), 9 (Cluster 2, 7.3%), and 16 (Cluster 3, 12.9%) cases, respectively. Key characteristics are summarized below:

Cluster 0—hyperkinetic + family stress Dominated by F90.0/F90.1 with Z63.5; mean diagnostic load 2.8 codes; highest co-morbidity.

Cluster 1—mixed presentation Widest diagnostic spread but lowest load (1.0 code); highest contextual adversity score (0.76).

Cluster 2—anxiety dominant Small, homogeneous group (8/9 carry F41.x); mean age ≈ 17.1 y.

Cluster 3—neurodevelopmental Enriched for F81 and F84.x; youngest mean age (≈14.9 y); diagnostic load 3.0 codes.

These cluster profiles demonstrate clear clinical phenotypes: Cluster 0 represents complex cases requiring intensive family-system interventions, Cluster 1 captures heterogeneous presentations with significant environmental stressors, Cluster 2 identifies an anxiety-focused subgroup amenable to targeted psychological interventions, and Cluster 3 highlights younger patients with neurodevelopmental needs requiring educational support.

In summary, the strongest organizational axis in this justice-involved sample is a hyperkinetic–family-stress phenotype, with distinct anxiety and neurodevelopmental subtypes emerging once Z-codes are incorporated.

### 3.4. Analysis of Clinician Recommendations

We first extracted the most frequent unigrams from the clinicians’ free-text recommendations using a CountVectorizer (stop words removed, terms appearing in at least 5 documents). The top 15 words are shown in Figure 1, with “parent” (83 occurrences), “counseling” (82), “psychoeducation” (65), “guidance” (59), “vocational” (59), and “psychotherapy” (47) leading the list.

Next, we applied Non-negative Matrix Factorization (NMF) to the recommendation corpus (n = 124 documents), specifying 5 topics. Table 1 lists the top terms for each topic, which we interpreted as follows:

- Topic 1: Vocational guidance and coaching support
- Topic 2: Parent counseling and maternal support
- Topic 3: Psychoeducational interventions
- Topic 4: Family-based psychotherapy
- Topic 5: Child-focused psychiatric follow-up, including pharmacotherapy

Linking topics back to diagnostic clusters, we observed that Cluster 0 (the hyperkinetic–family stress group) received disproportionately more Topic 2 and Topic 4 recommendations, underscoring clinicians’ emphasis on family-centered support for these cases. Older adolescents more frequently received vocational guidance (Topic 1), while psychoeducation (Topic 3) and psychiatric follow-up (Topic 5) were evenly spread across ages. Finally, F90.x codes were most often paired with Topics 2 and 4, F41.x with Topics 3 and 5, and Z-codes with Topics 1 and 2—demonstrating clinicians’ tailoring of interventions to specific diagnostic patterns.

## 4. Discussion

Our data-driven exploration demonstrates that routinely collected electronic information can illuminate clinically meaningful patterns even in a small, delinquency-oriented adolescent cohort. The systematic analysis of diagnostic frequencies revealed distinct patterns that align with both clinical experience and emerging research on justice-involved youth. Ranking ICD-10 frequencies confirmed that hyperkinetic disorders (F90.0–F90.1) together with family-centered Z-codes (e.g., Z63.5) dominate the diagnostic landscape, representing nearly half of all assigned diagnostic codes in our sample. This predominance is particularly striking when compared to general population prevalence rates and suggests that these adolescents face a dual burden of neurobiological vulnerabilities and environmental stressors. The co-occurrence of attention-deficit/hyperactivity symptoms with family disruption factors points to complex, multifaceted clinical presentations that require comprehensive intervention approaches. Furthermore, the clustering analysis revealed that these diagnostic patterns were not randomly distributed but rather formed coherent subgroups, with one cluster specifically characterized by the convergence of hyperkinetic disorders and family-related psychosocial stressors. This finding underscores the importance of examining comorbidity patterns rather than isolated diagnoses, as the interaction between individual vulnerabilities and environmental factors appears to shape distinct clinical phenotypes. The identification of such patterns through routine data analysis demonstrates the potential for transforming everyday clinical documentation into actionable insights that can guide resource allocation and treatment planning in adolescent mental health services.

### 4.1. Diagnostic Patterns and Comorbidity Insights

That cluster’s profile is similar to the “externalizing–disruptive” phenotype, which in a large genome-wide association analysis carries a distinct genetic signal [42]. Although our sample is modest and referral-based, such convergence implies that the unsupervised partitioning is not merely statistical noise but may tap into a biologically validated subtype. The prominence of F90 codes in our cohort also contrasts with national registry data, where only 9.5% of Finnish adolescent boys and 5.7% of girls held an ADHD diagnosis in 2023 [43]. This gap reinforces reports that justice-involved youth present with heightened hyperactivity and underscores why multimodal ADHD interventions should remain a core service priority.

The 44.7% prevalence of hyperkinetic disorders in our justice-involved sample starkly contrasts with general population rates of approximately 5–7% in adolescents, representing a 6–8 fold increase. This dramatic overrepresentation suggests that attention-deficit symptoms may serve as both a risk factor for and consequence of justice involvement, creating a bidirectional relationship between neurobiological vulnerabilities and environmental stressors. Regular monitoring of these diagnostic frequencies through automated quarterly reports could enable early detection of emerging mental health trends and inform resource allocation decisions.

The co-occurrence of F90.x and Z63.5 codes in our data extends beyond mere statistical association; it represents a clinically recognizable pattern of bidirectional influence between neurobiological vulnerabilities and family environmental stressors. Our finding that this comorbidity pattern emerges naturally from unsupervised clustering validates clinical intuition with data-driven evidence.

Beyond the dominant F90.x/Z63.5 pattern, our association analysis identified several other noteworthy comorbidities. The co-occurrence of F90.0 with Z62.2 (institutional upbringing issues) highlights how attention-deficit symptoms may be particularly challenging within structured educational environments. Similarly, the association between F90.1 and F81 (specific developmental disorders of scholastic skills) aligns with literature documenting the frequent overlap between hyperkinetic disorders and learning difficulties, suggesting a compound disadvantage requiring integrated educational and behavioral interventions. The clustering results also reveal important insights about diagnostic complexity in this population. The mean of 1.9 diagnoses per patient, while modest, masks considerable heterogeneity. Patients in Cluster 0 averaged nearly three diagnoses each, suggesting that the hyperkinetic-family stress combination may serve as a nexus attracting additional comorbid conditions. This accumulation of diagnoses in specific subgroups has important implications for service intensity and treatment duration. Clinical teams working with these high-complexity cases may need to plan for longer engagement periods and more frequent multidisciplinary coordination.

The translation of these findings into clinical practice requires concrete implementation strategies. Cluster 0 patients (hyperkinetic-family stress) should receive integrated treatment protocols combining medication management, family therapy, and parent training programs. Cluster 1’s heterogeneous presentations suggest the need for comprehensive assessments and flexible treatment planning. Cluster 2’s anxiety focus indicates cognitive-behavioral interventions as first-line treatments, while Cluster 3’s neurodevelopmental profile requires collaboration with educational services and specialized learning support.

### 4.2. Topic Modeling and Treatment Recommendations

The five thematic clusters identified through topic modeling of clinician recommendations provide valuable insights into treatment approaches for this population. The prominence of parent-oriented terms (Topics 2 and 4) across the dataset emphasizes the centrality of family-based interventions even in adolescent treatment, consistent with evidence-based practices for this age group. The clear delineation between vocational/educational approaches (Topic 1), family-centered counseling (Topics 2 and 4), and psychiatric/medication approaches (Topic 5) reflects the multi-modal treatment paradigm necessary for complex presentations.

Particularly noteworthy is the alignment between diagnostic patterns and recommended interventions. The association between Cluster 0 (the hyperkinetic-family stress cluster) and family-centered recommendation themes (Topics 2 and 4) suggests an appropriate clinical response to the identified comorbidity. This alignment between unsupervised clustering of diagnoses and thematic analysis of treatment recommendations provides a form of internal validation, indicating that clinicians are intuitively recognizing and addressing these patterns even before their formal computational identification.

The age-related patterns in recommendation topics—with vocational guidance more common for older adolescents—reflect developmental appropriateness in treatment planning. This age-stratified approach aligns with established developmental frameworks that emphasize identity formation and future orientation as central tasks of late adolescence [23], suggesting that clinicians are attentive to developmental stages when formulating interventions. The patterns identified could also be translated into routine practice by informing screening, treatment allocation, and staff training. For example, integrating the hyperkinetic–family stress profile into routine screening may help identify high-risk youth earlier, while staff training can focus on multidisciplinary interventions combining pharmacological, family, and educational components. Such alignment between data-driven subgroups and actionable strategies can strengthen the bridge between computational findings and clinical practice.

### 4.3. Methodological Considerations

Methodologically, the four-cluster solution produced by K-Means (implemented in scikit-learn [41]) and visualized with t-SNE [23] aligns with external machine-learning studies. Spear et al. analyzed pediatric records with the same pipeline and likewise recovered a four-cluster structure with an ADHD-enriched subgroup [44]. Orchard et al. found a comparable hyperactivity-dominated profile using partition-around-medoids on Ontario survey data [45]. Agreement across datasets of very different scale and provenance strengthens confidence in our exploratory findings and illustrates how low-cost, reproducible workflows can yield actionable insights for frontline mental-health services.

Although the silhouette score for the four-cluster solution was moderate (0.468), this structure still provided clinically meaningful subgroups. In line with prior machine learning work in adolescent psychiatry, modest silhouette values are common when dealing with high-dimensional and heterogeneous diagnostic data. Importantly, the incorporation of Z-codes enhanced separation, suggesting that psychosocial context is critical to distinguishing latent subgroups even in a modest sample. We therefore interpret the four clusters as exploratory but informative groupings that highlight key comorbidity profiles.

The silhouette score of 0.468 for our four-cluster solution indicates moderate internal validation, suggesting real structure in the data rather than arbitrary partitioning. Our approach to dimensionality reduction through t-SNE preserves local relationships between data points, allowing clinically meaningful clusters to emerge despite the high-dimensional feature space created by one-hot encoding of ICD codes. This methodological choice prioritizes the discovery of subtle patterns over linear separability, which is particularly appropriate for psychiatric data where boundaries between diagnostic categories are often blurred.

The combination of multiple analytical approaches—frequency analysis, association rule mining, clustering, and topic modeling—provides methodological triangulation that strengthens the validity of our findings. When consistent patterns emerge across different analytical methods, we can have greater confidence in their robustness. For example, the prominence of hyperkinetic disorders and family stressors appears in frequency counts, association rules, and cluster compositions, suggesting a genuine and clinically significant pattern rather than a methodological artifact.

### 4.4. Limitations and Future Directions

Several limitations warrant caution. The data come from a single specialized unit, cluster labels were inferred from cross-sectional ICD coding rather than longitudinal outcomes, and high-dimensional one-hot features were analyzed in a low-n setting. Furthermore, while our clustering approach provides valuable insights into potential subgroups, these clusters should be validated with external data and longitudinal outcomes before being used to inform clinical practice.

The moderate sample size (n = 124) limits statistical power and the generalizability of findings. Larger, multi-site cohorts would enable more robust clustering and potentially reveal additional subtypes beyond the four identified here. The reliance on clinician-assigned ICD codes introduces potential diagnostic variability, as coding practices may differ between practitioners. Standardized assessments would provide more reliable diagnostic information, though at the cost of ecological validity in routine clinical data.

The cross-sectional nature of our analysis precludes examination of how diagnostic patterns and treatment recommendations evolve over time. Longitudinal studies could determine whether the clusters identified represent stable subtypes or transient presentations, and whether tailored interventions based on these clusters lead to improved outcomes. Future work should address these limitations through prospective designs with standardized assessments and outcome tracking.

Despite these limitations, our study demonstrates the value of applying machine learning techniques to routinely collected clinical data. The patterns identified—particularly the co-occurrence of hyperkinetic disorders with family stressors—have immediate clinical relevance for treatment planning and resource allocation in adolescent mental health services. The alignment between these data-driven patterns and existing clinical knowledge suggests that our approach can formalize and extend tacit clinical understanding, potentially improving diagnostic precision and treatment personalization.

In sum, frequent ICD-10 mapping coupled with t-SNE-aided clustering gives a rapid decision-support tool that pinpoints resource-hungry diagnostic groups, guides targeted interventions, and moves adolescent delinquency care toward precision medicine. By identifying specific comorbidity patterns and their associated treatment recommendations, our approach provides a framework for more personalized and effective mental health interventions for adolescents, particularly those involved in the juvenile justice system.

The subjectivity inherent in ICD coding represents an important limitation, as different clinicians may assign varying codes to similar presentations. This diagnostic variability could impact clustering outcomes and limit generalizability. Future implementations should consider incorporating standardized assessment tools alongside clinical diagnoses to enhance reliability. Additionally, ethical considerations regarding the use of machine learning on sensitive adolescent mental health data include ensuring data privacy, obtaining appropriate consent for secondary analyses, preventing algorithmic bias against vulnerable populations, and maintaining transparency in how automated insights influence clinical decisions.

## 5. Conclusions

This study demonstrates the transformative potential of applying machine learning techniques to routinely collected clinical data in adolescent mental health services. Through the integration of multiple analytical approaches—frequency analysis, association rule mining, K-Means clustering, and topic modeling—we have uncovered clinically meaningful patterns that extend beyond traditional diagnostic categories. Our findings reveal that adolescents in the juvenile justice system present with distinct comorbidity profiles, particularly the convergence of hyperkinetic disorders and family-related psychosocial stressors, which require tailored intervention strategies.

The data-driven approach employed in this study offers several key contributions to the field. First, the identification of frequent ICD-10 code patterns provides immediate practical value for resource allocation and service planning. The dominance of hyperkinetic disorders (F90.0/F90.1) and family stress factors (Z63.5) highlights priority areas for intervention development and staff training. Second, the K-Means clustering analysis with t-SNE visualization revealed four distinct patient subgroups, each with unique diagnostic compositions and treatment needs. This clustering approach moves beyond single diagnosis thinking toward a more nuanced understanding of how multiple factors interact to create distinct clinical phenotypes. Third, the topic modeling of clinician recommendations uncovered five coherent treatment themes that align with identified diagnostic patterns, validating the clinical relevance of our computational findings.

The methodological framework developed in this study provides a reproducible template for other mental health services seeking to leverage their existing data for quality improvement. By using open-source tools and standard data formats, we demonstrate that sophisticated analyses need not require extensive resources or technical infrastructure. The combination of structured diagnostic data with unstructured clinical text offers a more comprehensive view than either data source alone, illustrating the value of mixed-method computational approaches in healthcare analytics.

Our findings have immediate implications for clinical practice and policy. The high prevalence of comorbid hyperkinetic and family stress presentations suggests that integrated treatment models addressing both neurobiological and environmental factors should be prioritized in justice-involved youth services. The alignment between diagnostic clusters and treatment recommendations indicates that clinicians are already recognizing these complex patterns intuitively; our computational approach formalizes this clinical wisdom and makes it actionable for service design. Furthermore, the contrast between our cohort’s diagnostic profile and general population prevalence rates underscores the unique mental health needs of justice-involved adolescents, supporting arguments for specialized services rather than generic mental health interventions.

Looking forward, this work establishes a foundation for precision medicine approaches in adolescent mental health. Future research should expand on these findings through several avenues. First, longitudinal studies tracking outcomes across our identified clusters would validate whether these subgroups represent stable phenotypes with differential treatment responses. Second, the integration of additional data sources—such as educational records, social services involvement, and standardized assessment scores—could enrich our understanding of these complex presentations. Third, the development of real-time decision support tools based on these patterns could help clinicians identify high-risk profiles and select appropriate interventions more quickly and accurately.

In conclusion, our multi-method machine learning analysis transforms routine clinical documentation into actionable insights for improving adolescent mental health services. By identifying specific comorbidity patterns, treatment themes, and patient subgroups, we provide an evidence-based framework for more personalized and effective interventions. As mental health services increasingly embrace digital transformation, approaches like ours demonstrate how existing data can be leveraged to enhance clinical decision-making, optimize resource allocation, and ultimately improve outcomes for vulnerable adolescent populations. The convergence of clinical expertise with computational methods represents a promising path forward for addressing the complex mental health needs of justice-involved youth.

Future research should address several key areas to advance this field. Longitudinal studies tracking diagnostic trajectories and treatment outcomes across our identified clusters would validate their clinical utility. Integration of additional data sources, including standardized assessments, educational records, and social services involvement, could enrich cluster profiles and improve prediction accuracy. Development of real-time clinical decision support tools based on these patterns could help clinicians rapidly identify high-risk profiles and select evidence-based interventions. Finally, multi-site validation studies are needed to assess the generalizability of these findings across different juvenile justice and mental health settings, ultimately working toward precision medicine approaches that match adolescents to optimal interventions based on their specific comorbidity profiles.

## Figures and Tables

**Table 1:** Top terms for each of the 5 NMF topics extracted from clinicians’ recommendations.

Topic	Key Terms
1	vocational, guidance, coaching, psychosocial, support, mother, parent
2	parent, counseling, mother, art, therapy, father, speech, parents
3	psychoeducation, coaching, support, psychosocial, counseling, therapy, vocational, test
4	psychotherapy, family, parents, speech, therapy, based
5	child, psychiatric, follow, pharmacotherapy, counseling, parent, vocational, therapy

## Data Availability

The original contributions presented in this study are included in the article. Further inquiries can be directed to the corresponding author.
